# Malaria incidence and prevalence during the first year of life in Nanoro, Burkina Faso: a birth-cohort study

**DOI:** 10.1186/s12936-018-2315-4

**Published:** 2018-04-12

**Authors:** Hamtandi Magloire Natama, Eduard Rovira-Vallbona, M. Athanase Somé, Serge Henri Zango, Hermann Sorgho, Pieter Guetens, Maminata Coulibaly-Traoré, Innocent Valea, Petra F. Mens, Henk D. F. H. Schallig, Luc Kestens, Halidou Tinto, Anna Rosanas-Urgell

**Affiliations:** 1 Department of Biomedical Sciences, Institute of Tropical Medicine, 2000 Antwerp, Belgium; 2 Unité de Recherche Clinique de Nanoro, Institut de Recherche en Sciences de la Santé, Nanoro, BP 218, Burkina Faso; 3 Department of Biomedical Sciences, University of Antwerp, 2610 Antwerp, Belgium; 4 Department of Medical Microbiology-Parasitology Unit, Academic Medical Centre, 1105 AZ Amsterdam, The Netherlands; 5 Centre Muraz, Bobo Dioulasso, BP 390, Burkina Faso

**Keywords:** Malaria, Incidence, Prevalence, Infants, Burkina Faso

## Abstract

**Background:**

Infants are thought to be protected against malaria during the first months of life mainly due to passage of maternal antibodies. However, in high transmission settings, malaria in early infancy is not uncommon and susceptibility to the infections varies between individuals. This study aimed to determine malaria morbidity and infection during early childhood in rural Burkina Faso.

**Methods:**

Malariometric indices were determined over 1-year follow-up in a birth cohort of 734 infants living in Nanoro health district. Clinical malaria episodes were determined by passive case detection at peripheral health centres while asymptomatic malaria infections were identified during  4 cross-sectional surveys at 3, 6, 9 and 12 months of age. *Plasmodium falciparum* infections were detected by rapid diagnostic test and/or light microscopy (LM) and quantitative PCR (qPCR).

**Results:**

In total, 717 clinical episodes were diagnosed by qPCR over 8335.18 person-months at risk. The overall malaria incidence was 1.03 per child-year and increased from 0.27 per child-year at 0–3 months of age to 1.92 per child-year at 9–12 months of age. Some 59% of children experienced at least one clinical episode with a median survival time estimated at 9.9 months, while 20% of infants experienced the first episode before 6 months of age. The majority of the clinical episodes were attributable to microscopic parasitaemia (84.2%), and there was a positive correlation between parasite density and age (Spearman’s rho = 0.30; *P *< 0.0001). Prevalence of asymptomatic infections was similar at 3, 6 and 9 months of age (17.7–20.1%) and nearly 1.6 times higher at 12 months (31.3%). Importantly, gametocyte prevalence among the LM-positive study population was 6.7%, but increased to 10% among asymptomatic infections. In addition, 46% of asymptomatic infections were only detected by qPCR suggesting that infants below 1 year are a potential reservoir for sustaining malaria transmission. Both symptomatic and asymptomatic infections showed marked seasonal distribution with the highest transmission period (July to December) accounting for about 89 and 77% of those infections, respectively.

**Conclusions:**

These findings indicate high and marked age and seasonal-dependency of malaria infections and disease during the first year of life in Nanoro, calling for intensified efforts to control malaria in rural Burkina Faso.

## Background

Despite the scaling-up of malaria control measures in sub-Saharan Africa (sSA), malaria morbidity and mortality remains particularly high in some areas, especially among young children and pregnant women [1]. In 2015, an estimated 191 million cases of malaria occurred in Africa resulting in 394,000 deaths of which nearly 74% were among children under 5 years old [2].

Malaria burden among children under 5 years is well documented in sSA [2–8] with the majority of studies focusing on the prevalence of malaria in infants below 6 months (including congenital malaria) [9–18]. However, few epidemiological birth cohort studies have analysed malaria morbidity during the first year of life [19–21], and surveys screening asymptomatic infections (from which clinical episodes may derive) are scarce in infants. Importantly, the evaluation of currently recommended interventions such as intermittent preventive treatment in infancy with sulfadoxine–pyrimethamine (SP–IPTi) [22] requires baseline information on infection and diseases in infants below 1 year in order to evaluate its impact.

Although children in early infancy are thought to be protected from malaria by maternal antibodies acquired in utero [23–26] and fetal haemoglobin (HbF) [27, 28], increasing evidence shows that prevalence of disease is higher than previously thought. Studies in high transmission settings have shown that the prevalence of malaria (cases and/or infections) in neonates could reach 25–46% [9, 11, 13, 14], 20–36% in the first 6 months of life [15–17], and 52.1% during the first year of life [29]. In Benin and Cameroon, the proportion of infants who experienced at least one malaria episode of malaria in 12-months birth cohort studies was estimated at 35–44.5%, respectively [20, 21]. On the other hand, some infants may develop a resistant or a tolerant phenotype upon prenatal exposure to malaria parasites, which influences the development of malaria symptoms or the time to first infection/clinical episode leading to inter-individual variation in susceptibility to malaria in early childhood [30–33]. Due to the life-threatening risk of malaria during the first year of life, detailed up-to-date epidemiological information on the malaria burden in infants, including asymptomatic infections, are necessary to inform countries policy decisions.

In Burkina Faso, malaria in infants remains the first cause of attendance to health centres [2]. Epidemiological data on malaria available in the country rely mostly on reports from health facilities, which lack accuracy and do not account for asymptomatic parasite carriers. To the best of found knowledge, only one birth cohort study evaluating the long-term effects of insecticide-treated nets in newborns was conducted in Nouna (northwest of Burkina Faso) between 2000 and 2003. The study reported incidences of infections of 0.75 and 2.7 per child/year during the periods of 0–5 and 6–12 months, respectively [34]. Thus, there is limited epidemiological data on malariometric indices, age-dependency of parasite densities and seasonal variation of the infections occurring during the first 12 months of life.

The aim of the current study was to investigate malaria infections and morbidity during the first year of life and assess variability in susceptibility between individuals in the rural health district of Nanoro in Burkina Faso. Using a highly sensitive quantitative polymerase chain-reaction (qPCR), the incidence of clinical episodes in a longitudinal survey and the age-specific prevalences of asymptomatic infections through cross-sectional surveys were determined. In addition, these results were compared with parasite detection by expert light microscopy (LM) to determine the prevalence of sub-microscopic infections in both symptomatic and asymptomatic patients. Finally, differences in susceptibility to malaria between individuals in early childhood were characterized.

## Methods

### Study area

**Fig. 1:**
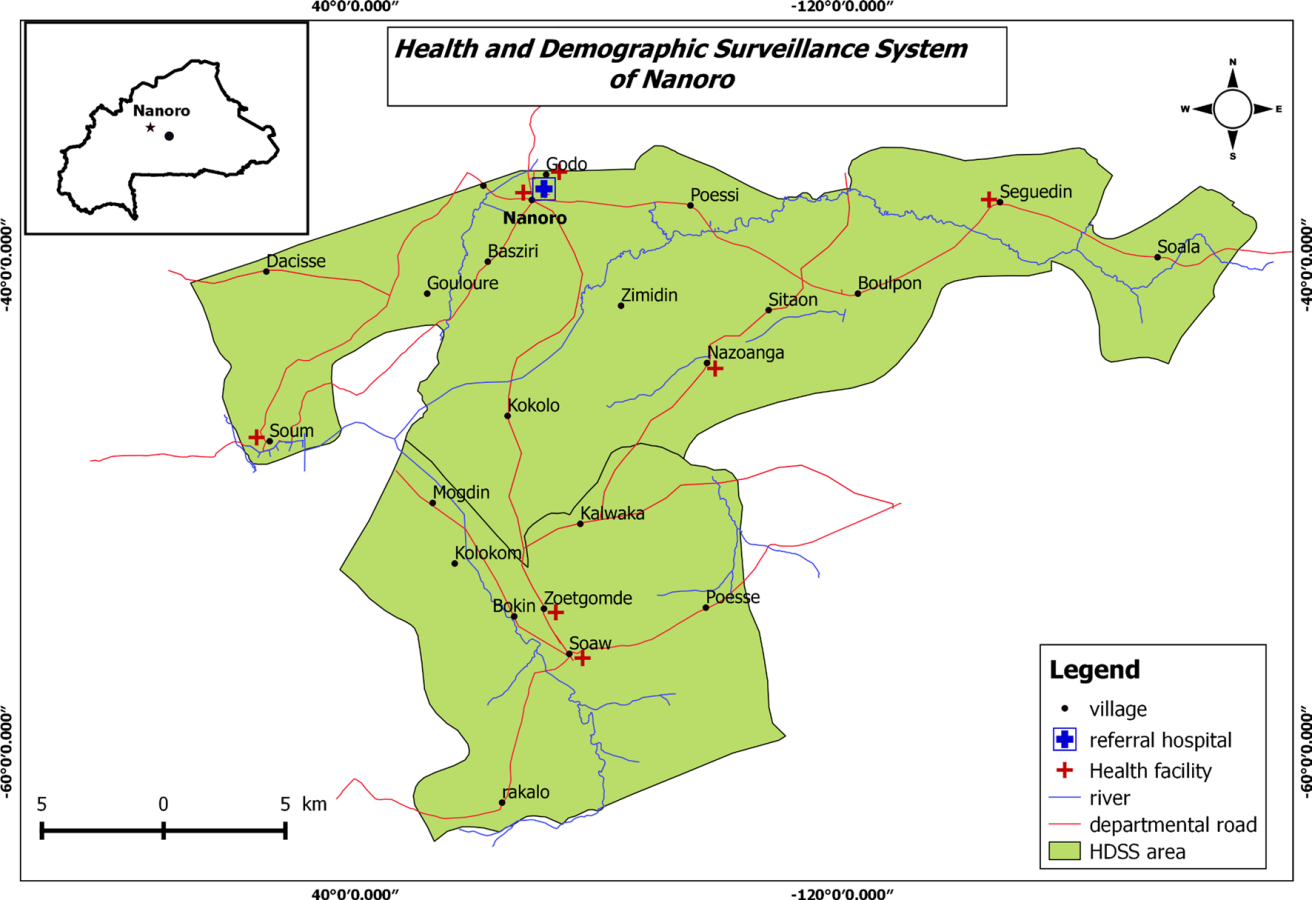
Map of the study area. Inset shows location of Nanoro Health District in Burkina Faso (black dot indicates location of capital Ouagadougou)

The study was conducted in Nanoro health district (NHD), a rural area in the central-west region of Burkina Faso at 85 km from Ouagadougou, the country’s capital (Fig. 1). NHD comprises 21 peripheral health centres and has approximately 166,683 inhabitants [35]. NHD is located in the Sudano-Sahelian area of the country with a rainy season lasting mostly from July to November with an average rainfall of 450–700 mm/year. Malaria transmission in the region is seasonal and hyperendemic. Almost all cases are caused by *Plasmodium falciparum* and the disease puts a significant burden on the population, especially in children under 5 years old and pregnant women. In 2016, health facilities in NHD reported 91,154 clinical malaria episodes, of which 9992 cases (11%) and 41,076 cases (45%) occurred among 0–1 year infants and 1–5 years children, respectively [35].

### Study design and procedures

This study was designed as a prospective birth cohort study with a 12-month follow-up duration of each newborn. The study was nested within the COSMIC clinical trial (NCT01941264), a multicentre, cluster-randomized trial assessing the effectiveness of community-based, scheduled screening and treatment of pregnant women for malaria control in pregnancy in Burkina Faso, Benin and The Gambia [36]. Pregnant women participating into the main trial in Burkina Faso were invited to enrol their offspring in the present study during antenatal care visits prior to delivery. Prior written informed consent was obtained from the mothers before inclusion in the study. Exclusion criteria were presence of major congenital malformation, chronic disease or signs of cerebral asphyxia. In total, 761 healthy newborns were enrolled over 16-months recruitment period (June 2014 to October 2015).

### Longitudinal follow-up

The longitudinal survey consisted of passive case detection of clinical episodes of malaria over 1 year. Mothers were encouraged to seek care any time their child felt sick at peripheral health centres where trained study nurses were appointed. At each visit, a clinical examination was performed and mothers reported previous health events. In the case of an axillary temperature ≥ 37.5 °C or history of fever within 24 h, a malaria rapid diagnostic test (RDT) was performed and positive infants were treated according to national guidelines (artemether–lumefantrine or artesunate–amodiaquine). Infants with severe malaria received either artesunate, artemether or quinine injection before being transferred to the *Centre Médical avec Antenne Chirurgical Saint Camille*, the referral hospital of NHD. From all febrile children, additional blood samples (blood smears and spots on Whatman 3MM filter papers) were collected from finger prick for retrospective analysis by LM and qPCR except for severe malaria cases for which LM results were made available. Infants with a negative RDT and those with no fever were further examined according to the national algorithm for the management of non-malaria illnesses in peripheral health centre. Free medications were provided for both malaria and non-malaria illnesses.

### Cross-sectional surveys

All children enrolled in the longitudinal follow-up were visited at home at 3, 6, 9, and 12 months of age to detect asymptomatic infections. Thus, only asymptomatic infants (i.e. no fever or history of fever in the previous 24 h) by the time of the survey were included. Each cross-sectional survey consisted of the administration of a questionnaire to mothers to collect previous heath events of their offspring related to malaria by a field worker and, the collection of blood samples from finger prick for LM and qPCR examination. Infants with ongoing malaria treatment were excluded for the survey. Those with fever or history of fever in the previous 24 h were referred to the peripheral health centre for the longitudinal survey.

### Detection of malaria infections

#### RDT

Malaria RDT was performed using SD-Bioline malaria antigen P.f^®^ test (05FK50, Standard Diagnostics, Inc, Korea) detecting *P. falciparum* histidine-rich protein2, as recommended by the National Malaria Control Programme (NMCP) in Burkina Faso. RDTs were performed following manufacturer instructions.

#### Microscopy

Malaria parasite detection and quantification by LM was performed according to standard procedures [37]. Briefly, blood smears were stained with Giemsa and examined with 100× oil immersion lens. For positive slides, the number of parasite and leucocytes were counted until reaching 200 leucocytes and parasitaemia was expressed as the number of asexual parasites per microlitre of blood based on an assumed 8000 white blood cells per microlitre of blood. A slide was considered negative if no parasites were observed after examining 100 fields. The presence of gametocytes was examined in all positive blood smears. All slides were read by two independent, experienced microscopists and those with discrepant results were read by a third microscopist. An internal quality control was performed by a fourth experienced reader for 10% of slides.

#### Filter paper processing, DNA extraction and VarATS quantitative PCR

Filter paper samples were air dried in the field, put in sealable bags with silica and transported the same day to the laboratory at the Clinical Research Unit of Nanoro (CRUN, Burkina Faso). The dried blood spots on filter papers were kept at ambient temperature until shipment to the Institute of Tropical Medicine (ITM, Belgium) for processing. Genomic DNA was extracted from 3 punches of dried blood spots (5 mm in diameter) using QIAamp 96 DNA blood kit (Qiagen, Germany), following the manufacturer’s recommendations and a final elution in 150 µL of water (Lonza, AccuGENE). Five µL of DNA were used as template for qPCR analysis targeting *P. falciparum var* gene acidic terminal sequence (varATS, ≈59 copies per genome) as previously described [38]. Parasite densities were obtained by interpolating cycle thresholds (Ct) using a standard curve prepared with titrated samples containing known numbers of infected erythrocytes diluted in whole blood (100,000–0.01 parasites/μL). The limit of detection of the varATS-based qPCR was 0.1 parasite/μL. Samples with Ct value > 39.7 were considered negative. DNA extracted from *P. falciparum* obtained from reference 3D7 parasites cultures was used as positive control. The negative controls included human negative blood spots on filter paper and mastermix reagent used as no template control (NTC).

### Case definitions

For the present analysis clinical malaria episode or symptomatic malaria infection was defined as the presence of *P. falciparum* of any density by qPCR and an axillary temperature ≥ 37.5 °C or history of fever within the past 24 h. Severe malaria was defined as a clinical episode with one or more danger signs of disease severity including impaired consciousness, respiratory distress, severe anaemia (haemoglobin concentration < 5 g/dL), incoercible vomiting and inability to drink or suckle [39]. Asymptomatic infections were defined as carriage of *P. falciparum* parasites detected by qPCR with no evidence of fever in the past 24 h. Sub-microscopic infections were defined as malaria infection or disease detected by qPCR but not by LM.

### Statistical analysis

Data were analysed with STATA version 12.0 (StataCorp, USA). Descriptive statistics were used to summarize baseline data and to compute malariometric indices. Kaplan–Meir analysis of time from birth to first clinical episode was used to determine the survival rates over the 12-months follow-up. The association between clinical malaria episodes, malaria transmission seasons and qPCR-parasite density were determined [odds ratios with 95% confidence interval (CI)] using bivariate logistic regression models. Spearman correlation coefficient was used to estimate the correlation between parasite densities and age of occurrence of clinical episodes. The difference in median values (with interquartile ranges, IQR) of parasite densities between (i) symptomatic and asymptomatic infections and (ii) infections occurring at 0–6 and 6–12 months, was analysed using the non-parametric Wilcoxon matched-pairs test. *P* values < 0.05 were considered statistically significant.

## Results

### Characteristics of study participants

Out of the initial 761 infants (739 singletons and 11 pairs of twins) enrolled, 669 (87.9%; 649 singletons and 20 twins) completed 1-year follow-up. Among the 92 (12.1%) infants who did not complete the follow-up, 7 (5 singletons and 2 twins from different mothers) died within 4 weeks after birth and 6 other singletons died before 12 months. In addition, 41 were lost to follow-up and 38 withdrew their consent mainly due to relocation out of the study area. Infants that were excluded from the analysis were the 7 neonates who died before 4 weeks of age due to the short duration of the follow-up and the 20 live twins owing to the risk of mixed samples and data from twin pairs.

Finally, 734 infants were included for the present analysis (Table 1). The mean age of their mothers was 26.4 ± 6.2 years and nearly two-thirds of them were multigravida (66.1%). More than half (62%) of pregnant women gave birth during malaria high-transmission season (from July to December); 48.5% of newborns were males and 51.5% females. The mean birth weight was 3012.7 ± 422.2 g. The proportions of asymptomatic infants enrolled into the cross-sectional surveys conducted at 3, 6, 9, and 12 months were 92.4% (678), 86% (631), 80% (587), and 86.9% (638), respectively. Out of the 649 singletons that completed the 12-months follow-up, 482 (74.3%) participated in the four cross-sectional surveys. Non-inclusion into the surveys was mainly attributable to ongoing malaria treatment, ongoing fever episode or the temporary absence from home. The highest number of infants excluded due to ongoing fever or history of fever in the previous 24 h was found at the cross-sectional survey performed at 9 months of age (2% [15/734]).

**Table 1:** Characteristics of study participants

Characteristics	Total study cohort (N = 734)
Maternal characteristics	
Age (years, mean ± SD)	26.4 ± 6.2
Gravidity [number (%)]	
Primigravida	132 (18.0)
Secundigravida	117 (15.9)
Multigravida	485 (66.1)
Insecticide treated net use [number (%)]	574 (78.2)
Infant characteristics	
Gender [number of females (%)]	378 (51.5)
Birth weight (g, mean ± SD)	3012.7 ± 422.2
Low birth weight (< 2500) [number (%)]	60 (8.2)
Birth season [number in malaria high-transmission season (%)]	455 (62.0)

*SD* Standard deviation, *g* gram

### Clinical malaria episodes

**Fig. 2:**
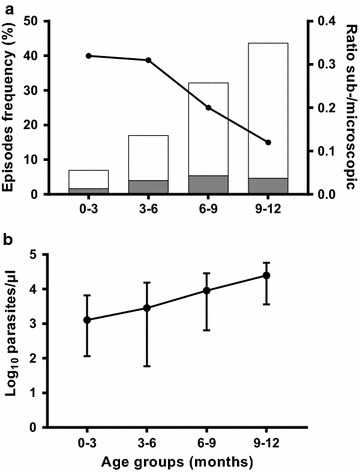
Clinical malaria episodes during the first year of life. **a** Frequency of clinical episodes by age group stratified by microscopic (white bars) and sub-microscopic (grey bars) malaria episodes. Black circles indicate the sub-microscopic:microscopic ratio. **b** Median parasite density (qPCR) on a log-scale by age group with interquartile ranges

In total, 717 clinical episodes of malaria were diagnosed by qPCR out of 1522 fever cases (47.11%) over 8335.18 person-months at risk, resulting in an incidence of malaria episodes during the first year of life of 1.03 per child-year. The proportion of clinical episodes increased from 7% (50/717) at 0–3 months to 43.8% (314/717) at 9–12 months (Fig. 2a and Table 2).

**Table 2:** Malariometric indices of clinical and asymptomatic infections during the first year of life

	Age groups				Total
	0–3 months	3–6 months	6–9 months	9–12 months	
Longitudinal survey (N = 734)					
Clinical episodes by qPCR [number (% out of total cases)]	50 (7)	122 (17)	231 (32.2)	314 (43.8)	717 (100)
Total time at risk (person-months)	2197.07	2124.91	2056.62	1956.58	8335.18
Incidence (per child-year)	0.27	0.69	1.35	1.92	1.03
Parasite density by qPCR [median (IQR)]	1294 (116.5–6726)	2848 (79.1–15,356)	9044 (642–28,554)	24,823 (3607–57,492)	10,826.5 (876–38,021)
Sub-microscopic infections [number (% out of cases per age group)]	12 (24)	29 (23.8)	39 (16.9)	34 (10.8)	114 (15.9)

	Surveys timepoints				Total
	3 months	6 months	9 months	12 months	
Cross-sectional surveys					
Total participants per survey	678	631	587	638	2534^a^
Asymptomatic infections by qPCR [number (%)]	120 (17.7)	127 (20.1)	108 (18.4)	200 (31.3)	555 (21.9)^b^
Parasite density by qPCR [median (IQR)]	148 (4.2–1754)	24.9 (1–954.7)	454.9 (3.1–4524)	1288 (2.6–13,998)	347.8 (1.94–3534)
Sub-microscopic infections [number (% out of cases per survey)]	58 (48.3)	69 (54.3)	49 (45.4)	79 (39.5)	255 (45.9)

*IQR* interquartile range

^a^ Total samples screened for asymptomatic infections

^b^ Proportion of qPCR-positive samples out of total screened over the four cross-sectional surveys

A total of 433 children (59%) experienced at least one clinical episode, and 201 (27.4%) experienced more than one episode. The mean number of clinical episode was 1.6 (range 1–4) per child. Nearly two-thirds (65.6% [284/433]) of the first malaria episodes occurred during the period of 6–12 months of age as compared to one-third (34.4% [149/433]) during the period from birth to 6 months. The survival analysis shows that the 6-months survival rate was 80%, while half of the infants (50% [367/734]) experienced the first clinical episode by 9.9 months. Severe malaria episodes represented 1.5% of total clinical cases. The frequencies of danger signs of severe malaria were: incoercible vomiting (63.6% [7/11]), inability to drink or suckle (36.4% [4/11]), impaired consciousness (27.3% [3/11]), respiratory distress (18.2% [2/11]), and severe anaemia (18.2% [2/11]).

Overall, parasite densities by qPCR correlated with age (Spearman’s rho = 0.30; *P *< 0.001), with a median increasing from 1293.75 [IQR 127.00–7606.75] parasites/µL for 0–3 months to 24,822.50 [IQR 3624.00–57,650.25] parasites/µL for 9–12 months of age (Fig. 2b). Clinical episodes occurring from 6 to 12 months of age had 6.4 times higher parasite densities than those detected during the first 6 months of life (15,923.50 [IQR 1644.20–45,217.50] versus 2499.75 [IQR 85.00–14,230.50], *P *< 0.001).

The majority of clinical episodes presented with parasite densities detectable with LM (603/717, 84.1%). The distributions of sub-microscopic and microscopic parasitaemia according to age is presented in Fig. 2a. The proportion of sub-microscopic infections decreased with age as illustrated by the sub-microscopic:microscopic ratio, which decreased from 0.32 to 0.12 in infants from 0–3 to 9–12 months of age, respectively. The risk of having a sub-microscopic clinical episode was 1.8 times higher during the first 6 months of life (41/172, 23.8% sub-microscopic rate) than that during the period of 6–12 months of age (73/545, 13.4% sub-microscopic rate, risk ratio = 1.8; 95% CI = 1.28–2.54; *P *= 0.001).

### Asymptomatic infections

**Fig. 3:**
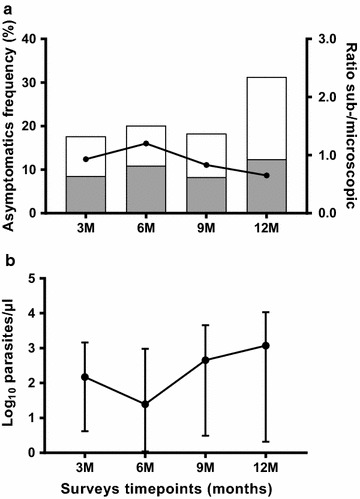
Asymptomatic malaria infections during the first year of life. **a** Prevalence of asymptomatic infections by age, stratified by microscopic (white bars) and sub-microscopic (grey bars). Black circles indicate the sub-microscopic:microscopic ratio. **b** Median parasite density (qPCR) on a log-scale by age with interquartile ranges

The prevalence of asymptomatic infections detected by qPCR were similar at 3, 6 and 9 months of age (120/678, 17.7%; 127/631, 20.1%; 108/587, 18.4%, respectively) but higher at 12 months (200/638, 31.3%) (Fig. 3a, Table 2). Among infants who completed the four surveys (N = 482), 156 (32.4%) experienced at least one asymptomatic infection, and 100 (20.7%) had at least two asymptomatic infections. The mean number of asymptomatic infections acquired per child was 1.5 (range 1–4). From all infants who completed the 12-months follow-up and did not develop any clinical malaria episode (N = 241), 104 (43.1%) had an asymptomatic infection at least in one survey.

By qPCR, the overall median parasite density was 347.8 [IQR 1.94–3534] parasites/µL, which was significantly lower than that of clinical episodes (10,826.5 [IQR 876–38,021], *P *< 0.001). As shown in Fig. 3b, there was a tendency of increased parasite density in asymptomatic infections from 6 months of age. The median of cumulative parasite density at 3 and 6 months surveys was significantly lower than that observed at 9 and 12 months surveys: 87.5 [IQR 1.61–1262.5] parasites/µL versus 819.63 [IQR 2.2–7584] parasites/µL, *P *< 0.001.

In total, 46% (255/555) of asymptomatic infections were only detected by qPCR. The ratio of sub-microscopic:microscopic infections decreased from 0.9 to 1.2 at 3 and 6 months surveys to 0.6 at 12 months surveys (Fig. 3a).

### Seasonality of *Plasmodium falciparum* infections

**Fig. 4:**
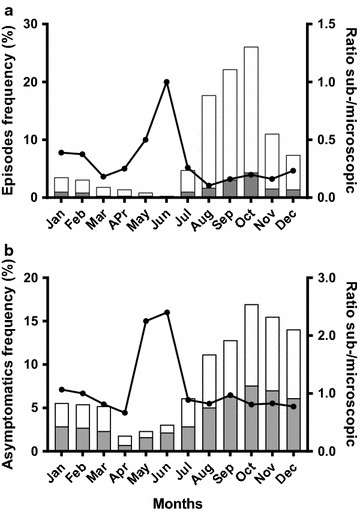
Monthly distribution of malaria infections during the first year of life. **a** Clinical episodes; **b** asymptomatic infections, stratified by microscopic (white bars) and sub-microscopic (grey bars) infection. Black circles indicate the sub-microscopic:microscopic ratio

**Fig. 5:**
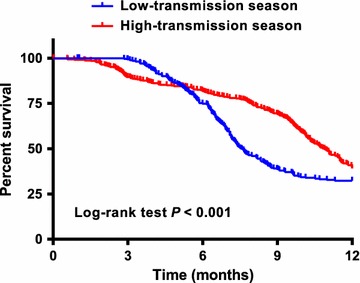
Risk of clinical malaria episode during the first year of life according to birth season. Kaplan–Meier survival curves stratified by infants born during malaria high-transmission season (July–December, red line) and low-transmission season (January–June, blue line)

Both clinical episodes and asymptomatic infections showed a marked seasonal pattern (Fig. 4). The majority of clinical episodes (639/717, 89.1%) and asymptomatic infections (425/555, 76.6%) were diagnosed from July to December corresponding to the wet season with a peak in October. The odds ratio of having a clinical episode during the high-transmission versus the low-transmission season was 7.01 (95% CI 5.4–9.1; *P *< 0.001). The lowest frequencies of malaria episodes were observed in May and June (6/717, 0.8% and 2/717, 0.3%, respectively) (Fig. 4a). Infants born during malaria high-transmission season were at higher risk of experiencing a first clinical episode during the first 6 months of life whereas their counterparts born during malaria low-transmission season had a higher risk from 6 to 12 months of age (Fig. 5). Overall, the risk of having an asymptomatic infection was significantly higher when the cross-sectional survey occurred during the malaria high-transmission season from July to December (Table 3). The monthly distribution of asymptomatic infections showed that the beginning of the dry season (January to March) was characterized by a high number of asymptomatic infections (range 5.2–5.8%) compared to the period from April to June (range 1.8–3.1%) (Fig. 4b). The evolution of sub-microscopic:microscopic infections ratios by month highlighted a year-round higher ratios in asymptomatics [mean 0.9, range 0.7 (April)–2.4 (June)] than in clinical episodes [mean 0.2, range 0.1 (August)–1 (April)]. The ratios in asymptomatics compared to symptomatic infections, were 3.2 times higher during malaria low-transmission season (1.13 versus 0.35, respectively) and nearly 5 times higher during malaria high-transmission season (0.84 versus 0.17, respectively) (Fig. 4).

**Table 3:** Age-specific risk of asymptomatic infections in the context of seasonal risk of infection in Burkina Faso

Month of birth	Surveys timepoints							
	3 months		6 months		9 months		12 months	
	OR (95% CI)	*P*	OR (95% CI)	*P*	OR (95% CI)	*P*	OR (95%CI)	*P*
Q1 (January–March)	Ref		Ref		Ref		Ref	
Q2 (April–June)	*22.05 (2.90–167.78)*	*0.003*	*2.30 (1.27–4.15)*	*0.005*	0.72 (0.39–1.33)	0.298	0.70 (0.36–1.37)	0.298
Q3 (July–September)	*88.96 (12.27–645.15)*	*< 0.001*	1.22 (0.71–2.11)	0.464	*0.14 (0.07–0.30)*	< *0.001*	*2.13* (*1.25–3.61*)	*0.005*
Q4 (October–December)	7.47 (0.96–58.30)	0.055	*0.15 (0.06–0.37)*	*< 0.001*	0.64 (0.38–1.10)	0.105	*3.66* (*2.15–6.22*)	*< 0.001*

*Q* quarter, *OR* odds ratio, *CI* confidence interval, *Ref* reference, OR (95% CI) with P values (*P*) < 0.05 are shown in italic

### Gametocyte carriage

The proportion of clinical episodes carrying gametocytes among LM-positive samples was 3.6% (22/603), with the 22 cases occurring in different individuals. Out of the 300 LM-positive asymptomatic infections, *P. falciparum* gametocytes were detected in 29 blood smears from 28 individuals (9.7%). In total, 49 different gametocyte carriers were detected at both cross-sectional and longitudinal survey, resulting in an overall gametocyte prevalence of 6.7% (49/734) among the study population. The median gametocyte density was significantly higher in asymptomatic infections than in clinical episodes (128 [IQR 72–286] versus 48 [IQR 32–127] parasites/µL, *P *= 0.011).

## Discussion

This study describes malaria infection and morbidity during the first year of life in NHD, a rural area of Burkina Faso where the seasonal malaria chemoprevention (SMC) in children and SP–IPTi were not implemented at the time the study was conducted, though adopted by NMCP in 2014 [39]. The findings showed a high burden of *P. falciparum* infections, which was characterized by an overall incidence of clinical malaria of 1.03 per child-year, an age-specific prevalence of asymptomatic infections ranging from 17.7 to 30.4% by cross-sectional survey, and a significant number of cases occurring during the first 6 months of life.

This high prevalence and incidence of infections and disease is in agreement with previous studies in the country [8, 34] and other reports from sSA [16, 18, 29, 40, 41], but contrasts with the previous perception of malaria as an uncommon infection/disease in early infancy in endemic settings [42–44].

The acquisition of maternal antibodies due to transplacental transfer and the presence of HbF in early infancy [23–28] are considered to provide a certain degree of protection against malaria to newborns during the first 6 months of life. Although there was a significant difference in both clinical and asymptomatic *Plasmodium* infections between 0–6 and 6–12 months, about 29% of infants experienced a clinical episode and/or asymptomatic infection during the first 6 months of life. On the other hand, the observed increase of malaria morbidity with age is in agreement with findings of a pooled analysis, which included data from different epidemiological settings in sSA [45].

The majority of clinical episodes had parasite densities detectable by LM (84%), while 46% of asymptomatic infections carried parasite densities only detectable by qPCR, indicating that parasite levels in peripheral blood are associated with clinical manifestations in infants. Moreover, in agreement with previous reports [29], parasite densities increased with age both in symptomatic and asymptomatic infections, with parasite densities from 0 to 6 months significantly lower than those from 6 to 12 months. That age-dependency of parasite load was further illustrated by a ratio of sub-microscopic: microscopic malaria infections decreasing after 6 months of age (Figs. 2 and 3). Altogether, these results indicate a gradual increase in malaria susceptibility during the first year of life as the passive immunity acquired from the mothers and levels of HbF progressively fade [28, 46–48]. Importantly, although gametocyte carriage could not be assessed here by reverse transcriptase (RT) qPCR, a prevalence of gametocyte of 6.7% was found among the study population, which increased to approximately 10% among LM-positive infants with asymptomatic infections. Therefore, the high rates of asymptomatics (and eventually sub-microscopic infections) found in the current study highlights the potential contribution of this age group as a reservoir for malaria transmission. Indeed, a previous study conducted in Burkina Faso has shown that sub-microscopic infections account for nearly 30% of human–mosquito transmission in the country, with a prominent contribution of children over adults to the infectious reservoir [49].

A marked seasonal distribution of malaria cases and infections was also observed, with the highest transmission period (coinciding with the wet season from July to December) accounting for 89 and 77% of all clinical episodes and asymptomatic infections respectively. In this regard, strategies such as SMC in children aged 3–59 months (from August to November) [39] can have an important impact in reducing the malaria burden during the rainy season. However, 23% of all asymptomatic infections (53% of which are also sub-microscopic) occur during the dry season, when SMC is not provided. In this context, SP–IPTi, which is administrated at 3, 4 and 9 months of age following the expanded programme for immunization schedule, can be a crucial strategy to clear asymptomatic infections irrespective of the transmission season, thus avoiding asymptomatic infections to further develop into clinical cases and/or to sustain transmission. Of note, 0.23 clinical malaria episodes per child-year occurred during the first 3 months of life and may be due to new infections or, during the first weeks of life, due to congenital transfer [13, 50–53] with potential life-threatening consequences for the infant at the long-term [53–55]. Currently, there is a lack of particular strategies to specifically target this age group, while the incidence and prevalence of cases and infections together with malaria deaths in this age group are urgently calling for new or improved preventive strategies as well as case management guidelines. In this sense, it could be that additional emphasis on vector control is merited, going for balance between improved drug-based and non-drug based strategies and, also addressing transmission reduction in addition to disease burden reduction.

In this study, 1.5% of infants developed severe malaria, which was lower than that estimated in peripheral health centres (4.2%) between 2014 and 2016 in the central-west region of the country [35, 56]. Although clinical episodes were passively detected in the present study, there was a close follow-up of infants through cross-sectional surveys and sensitization of the mothers for early health seeking for any health issue. Early diagnosis and treatment of clinical malaria episodes has probably contributed to the reduction of severe malaria cases and related-mortality among the study population [57, 58]. Owing to the recent implementation by the Burkina Faso government of medication free of charge for infectious diseases (including malaria) for children under 5 years, a reduction of malaria mortality among infants is expected in the future. The challenge will be to maintain the availability of RDTs and malaria treatments in health centres across the country, which has been intermittent in the past, and to increase the number of health facilities in areas with low coverage.

Overall, the number of malaria episodes and asymptomatic infections vary among the study population with some infants not developing a malaria episode despite carrying parasite infections (asymptomatic infections) at one or several time points during the follow-up, suggesting a potential control or tolerance of malaria infections by some infants [30, 31]. Unfortunately parasite population genotyping, which would have allowed investigation of whether asymptomatic infections detected at cross-sectional surveys were linked to any disease burden, was not performed in the present study.

Variation at individual level could be partially explained by differential benefit of the protective effect of maternal antibodies as their levels may vary from one newborn to another [19, 23, 24, 26, 41, 59]. However, factors that modulate malaria risk/protection in early childhood with regards to the transmission seasonality are not fully understood and further and complementary studies addressing the effect of pregnancy preventive treatments [20, 60], in utero exposure to malaria parasites and/or antigens and subsequent modification of fetal immunity [61–65] and host genetic polymorphisms in specific genes [66–68] are needed.

## Conclusions

Despite substantial efforts made in malaria control during this current decade in Burkina Faso, this study showed a high burden of malaria infections, with marked age and seasonal-dependency, during the first year of life in Nanoro. The high rates of asymptomatic infections (with a gametocyte carriage of 10% among LM-positive cases) and of sub-microscopic infections suggest that infants under 1 year old are a potential reservoir for sustaining malaria transmission. Intensified efforts to control malaria in rural Burkina Faso are urgently needed in order to reduce the disease burden in susceptible populations.
